# Rehabilitation Evidence-Based Decision-Making: The READ Model

**DOI:** 10.3389/fresc.2021.726410

**Published:** 2021-10-05

**Authors:** Iona Novak, Anna te Velde, Ashleigh Hines, Emma Stanton, Maria Mc Namara, Madison C. B. Paton, Megan Finch-Edmondson, Catherine Morgan

**Affiliations:** ^1^Faculty of Medicine and Health, The University of Sydney, Sydney, NSW, Australia; ^2^Specialty of Child and Adolescent Health, Faculty of Medicine and Health, Sydney Medical School, The Cerebral Palsy Alliance Research Institute, University of Sydney, Sydney, NSW, Australia

**Keywords:** evidence-based, decision-making, READ model, rehabilitation, goal-setting

## Abstract

Evidence-based practice is the foundation of rehabilitation for maximizing client outcomes. However, an unacceptably high number of ineffective or outdated interventions are still implemented, leading to sub-optimal outcomes for clients. This paper proposes the Rehabilitation Evidence bAsed Decision-Making (READ) Model, a decision-making algorithm for evidence-based decision-making in rehabilitation settings. The READ Model outlines a step-by-step layered process for healthcare professionals to collaboratively set goals, and to select appropriate interventions. The READ Model acknowledges the important multi-layered contributions of client's preferences and values, family supports available, and external environmental factors such as funding, availability of services and access. Healthcare professionals can apply the READ Model to choose interventions that are evidence-based, with an appropriate mode, dose, and with regular review, in order to achieve client's goals. Two case studies are used to demonstrate application of the READ Model: cerebral palsy and autism spectrum disorder. The READ Model applies the four central principles of evidence-based practice and can be applied across multiple rehabilitation settings.

## Introduction

Evidence-based practice (EBP) has become the cornerstone of care to maximize client outcomes through the application of best-available interventions. Despite this, clinical practice may lag as much as 10–20 years behind research (1). These translational “Valleys of Death,” i.e., the transition from basic research to clinical knowledge and from clinical knowledge to practice or implementation and then policy (2), are longer than a whole childhood and potentially harmful for pediatric rehabilitation clients. For example, a systematic review of preventable risk factors for Sudden Infant Death Syndrome (SIDS) published in 2005 found that earlier implementation of the supine sleeping “Back to Sleep” campaign, may have prevented at least 50,000 infant deaths in Europe, the USA, and Australasia since the 1970's (3). This evidence lag is also likely a significant underestimation, with studies only measuring part of the evidence-to-practice process (4). As well as a widespread lag in adoption of new treatments, only 55% of clients receive recommended treatments (5, 6) and another 43% receive care that is inappropriate or potentially harmful (7). Barriers to evidence uptake in rehabilitation are real and are known to include time, skills, confidence, institutional support, workflow discordant to the new evidence, and patient preferences that differ (8–10). In addition, the production of rehabilitation evidence is occurring at an even faster rate than pharmacology, meaning professionals experience information overload (11). Such challenges highlight the need for well-reasoned decision-making within EBP.

The core tenant of evidence-based decision-making in rehabilitation is to make a decision that achieves the best outcome for the client. EBP is underpinned by models of rationality, where our decisions, actions, and beliefs are a rational response to the trustworthiness of evidence (12). In addition to rationality, rehabilitation is underpinned by the biopsychosocial model that acknowledges the complex interaction of biological, psychological, and social factors on a person's health (13). As a consequence, evidence-based rehabilitation takes a step beyond trusting evidence to also placing a high value on person-centered or family-centered care, multidisciplinary teamwork, good communication, problem-solving, practice of functional activities, psychosocial support, and tailoring of intervention to meet the person's goals and needs (13). Decision-making in pediatrics is usually conducted by parents on behalf of the child, driven by the desire to try everything possible (14). This is complex because decisions will understandably be influenced by parental beliefs, hopes, sorrow, and values (14–16). The Life Needs Model and experts in the field have proposed several key principles for evidence-informed decision-making in rehabilitation. These include family-centered practice where the family is the decision-maker, shared decision-making, strengths-based (focusing on what the child *can* do), recognizing intrinsic worth and dignity, goal setting, deliberative consideration and discussion, and fostering equality (17–19).

Although recent surveys indicate some clinicians use evidence in pediatric rehabilitation (20, 21), there is still an unacceptable use of ineffective interventions and outdated care (22, 23). Multifaceted knowledge translation interventions are known to help rehabilitation professionals bridge the research-to-practice gap (24). The understanding of EBP often, regrettably, gets reduced to a description of treatment benefits from randomized controlled trials. This reductionist practice has drawn criticism from experts because it fails to pay enough attention to the original definition of EBP, which equally includes the preferences of the family (individual or caregivers) and the clinical context, including judgement of the clinical practitioner (25). Within the pediatric rehabilitation field, the volume and quality of intervention trials is comparatively low and important evidence from other research designs might not be given the attention they deserve (18, 26). This is further complicated by the heterogeneity of disability even within well-defined conditions such as stroke, autism spectrum disorder, and cerebral palsy. Differences in type, topography, severity, and associated impairments of any condition mean that interventions with evidence cannot be applied equally.

The aim of this paper is to propose a model for comprehensive evidence-based algorithmic decision-making in rehabilitation settings. This incorporates all three central principles of EBP and addresses the concerns of critics, with the aim of improving client outcomes.

## Methodology

The model was developed from systematic review evidence about effective: evidence-based practice implementation (8–11); shared decision-making (18); decision-aides (27); and evidence-based algorithms for decision-making (28). Coupled with authoritative perspectives on best-practice rehabilitation (13); value-based medicine (29); and “P5 medicine” (predictive, personalized, preventive, participatory, and psycho-cognitive medicine) (29). Our model is underpinned by key theoretical frameworks from the rehabilitation literature of goal setting, person-centered care, rational decision-making, whilst acknowledging, and responding to a person's biopsychosocial complexities and the whole of life needs. The authors are a team of clinicians (*n* = 2), clinician-scientists (*n* = 4), and scientists (*n* = 2), who work in a not-for-profit community-based rehabilitation service that provides rehabilitation to over 6,000 clients annually with a range of neurological disabilities both childhood and adult onset, and have conducted research in the field of evidence-based practice implementation and decision-making (24, 30, 31).

## Read Model

Layering is a concept applied in photo editing software for the post processing of digital photographs. Described as “non-destructive editing techniques to add color or dimension, without changing the original photo information” (32), layering is the freedom to finesse and sculpt a photo, and each layer can be customized depending on the desired outcome. When a digital photograph is edited, the aim is to enhance focal points, minimize the ambiguity of shadows, and illuminate gray areas. The layers are not stand-alone editing techniques, but rather integrated and interdependent. Taken together, they improve the precision and merit of the picture.

**Figure 1:**
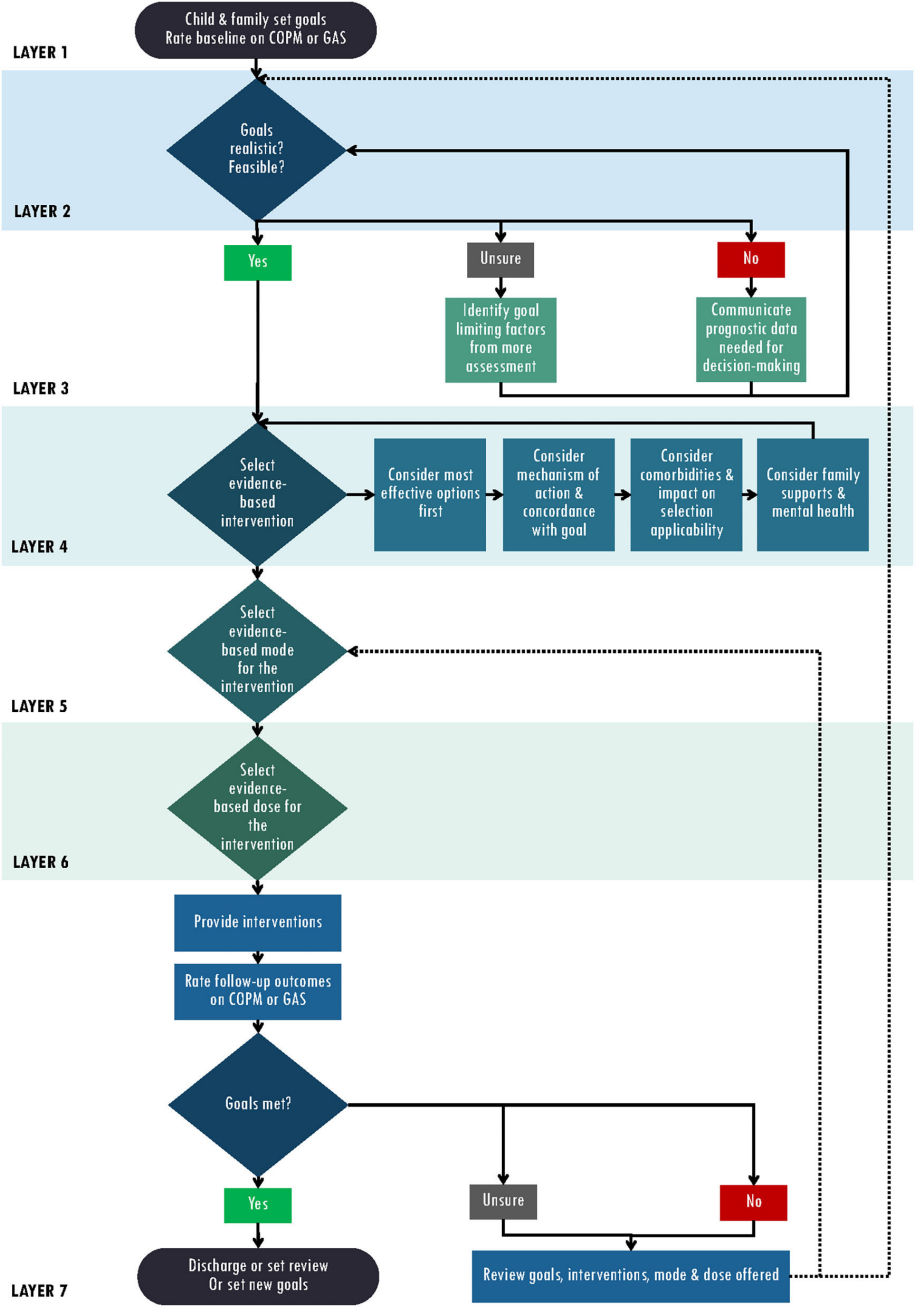
READ model.

Evidence-based decision-making as a process, can be compared to the process of layering in photo editing. We propose a model of layered evidence-based decision-making entitled Rehabilitation Evidence bAsed Decisions (READ Model) (Figure 1). The model begins with Layer 1 Child and family set baseline goals. This first layer can be likened to the original photograph; goal setting is the recommended starting place for all healthcare professionals conducting evidence-based intervention, and it should always be present and prominent throughout the READ Model. Layer 2 builds upon the first layer, where healthcare professionals bring into focus if a goal is realistic, feasible, and achievable, ensuring full transparency of communication and service planning. Layer 3 involves considering whether the client's goals are realistic given available interventions and the client's level of disability. The intervention/mechanism of action should match the goal, as well as take into account the client's comorbidities, family supports and other external factors. Next the type, mode and intensity (Layers 4–6) of the intervention to target the chosen goal will depend on the child; and can be layered in different amounts and delivery formats as chosen by the health care professional, child, and family informed by evidence. Layer 7 is the final layer and includes the delivery and evaluation of the chosen intervention, with steps recommended to re-visit a layer when goals are not achieved including revising and setting alternative goals, or trialing alternative interventions, modes, or intensities of intervention in order to achieve the goal. Together, these layers create a comprehensive decision-making framework that seeks to optimize client outcomes.

### READ Model Layer 1: Child and Family Set Goals for Intervention

Framing the entire focus of intervention through the lens of child-led or parent-set proxy goals is best practice in rehabilitation and reflects the latest evidence. We recommend starting with goal setting in this model and be aware that adjustments may need to be made in Layers 1–3. In accordance with goal-setting theory, encouraging the child and family to set goals increases attention, effort, motivation and behavioral intent toward intervention targets, and in-turn helps to produce better clinical outcomes as well as persistence (33). Goal-focused interventions also address client concerns and result in meaningful therapeutic improvements. Goals are central because key principles of neuroplasticity are harnessed through goal directed practice of real-life tasks, intense repetition, and ensuring the “just right” challenge (34). Children are likely to practice a goal more often and more intensely when they are motivated to learn the new task. Therefore, having a greater understanding of a child's motivations may improve the goal setting and intervention planning process, which may lead to improved therapy outcomes (35).

We recommend the use of standardized, evidence-based goal-setting, and evaluative instruments to illicit and measure progress of an intervention, as well as to promote independence. Standardized evaluative instruments also assist with aggregation of data across diagnostic groups and service delivery formats for measuring quality assurance, efficiency, efficacy, and cost effectiveness. The Canadian Occupational Performance Measure (COPM) is a widely recognized, standardized goal setting, and outcome measure (36). The COPM enables a child or parent proxy to rate the child's performance of the goal and satisfaction with performance on a scale of 1–10 to measure perceived change over time. The Goal Attainment Scale (GAS) is an individualized criterion-referenced measure of goal performance (37) with good to excellent reliability in rehabilitation settings (38, 39). The goals are set with five possible outcomes which are used to measure a child's change in performance following the intervention. The GAS is similar to the COPM but because there are five possible outcomes, it has even greater responsivity. Both the COPM and GAS are feasible and reliable tools which can be used to measure the effectiveness (with responsivity to change) of therapy interventions across all age ranges, diagnoses, and severities (40). Selection of one, or both, of these appropriate outcome measures can help to confirm whether an intervention is both effective and can assist the child and family achieve their goals.

### READ Model Layer 2: Are the Goals Realistic? Are the Goals Feasible for Our Service?

Complications may arise in the goal setting process when hopes and reality collide. When unachievable goals are set for any reason, dissatisfaction inevitably ensues. Dissatisfaction with rehabilitation providers or therapy can lead to gradual disengagement from rehabilitation in general, which may contribute to deterioration in function. Setting goals that are achievable and realistic often involve honest and difficult conversations with children and their families. It is important that the gap between the hopes of the family and the reality of the situation is met with compassion and empathy, and validation of the hopes themselves (41). Opening the conversation to a discussion about what the family thinks will happen next in their child's trajectory can assist in developing short term goals In addition, use of the International Classification of Functioning, Disability, and Health (ICF) Core SETS can be used to shape the selection of meaningful goals from a comprehensive library in relevant domains to the child and parent (42). The core sets can provide guidance to professionals on how to tailor the goal setting process so as to individualize the rehabilitation to the person's context and external constraints (e.g., how to promote sleep hygiene within a noisy household). At times, professionals and families may disagree on goals and their importance, but in person-centered care the family is empowered to set the priorities and the professional's role is to provide informed guidance, not adjudicate.

The “SMART” framework has been applied across many fields and helps shape goals that are possible to achieve. SMART is an acronym for *Specific, Measurable, Achievable, Realistic/Relevant* and *Timed*. Constructing goals according to this framework helps develop a working partnership between the health professional/health service and the client/family. Even goals that are realistic for the individual might not be possible within the resources and context of the clinical service and these factors must feed into the goal setting process (43, 44).

If the goals are deemed both realistic and feasible, the next step is to sharpen the focus by selecting the appropriate intervention/s to match the goal. If there is still uncertainty, more assessment might be required including observation of the current level of performance of the task to determine if it is realistic. At this point, the aim of assessment is not to identify deficits at the body structures level, but to ascertain which of the goal limiting factors are treatable. If the service provider cannot adjust their service model to provide the best practice interventions for the goal to be attained (e.g., if a home visit is required but not permitted), then a referral elsewhere should be made.

### READ Model Layer 3: Communicate the Prognosis and Adjust Goals (Where Required)

Situations can arise where it is identified that the desired goals are not realistic. This is commonly related to a misunderstanding of the child's prognosis, and it is at this point that a conversation about prognosis must take place. This conversation should be informed by a thorough understanding of the diagnostic and prognostic evidence related to the client's condition, which will include important observational studies such as population register and case-control data. Guidance about how to shape this conversation is available, including the “SPIKES” protocol which outlines strategies to include when sharing difficult news (45). The ultimate aim of the conversation is to help parents understand their child's likely trajectory as well as provide information about where their child is currently on that trajectory and discuss where they might be heading. In cerebral palsy, a number of classification tools exist that can be useful in these prognostic conversations, and these are highlighted in the scenarios below.

Scenario 1: Charlie is 5 years old, has spastic quadriplegia and a cortical vision impairment. His parents have set a goal of walking independently. During his most recent physiotherapy sessions Charlie has been working on head control by sitting on a slowly moving ball and practicing lifting his head in prone, over a roller. To assess whether the goal of walking is realistic, the current team assess Charlie in detail with the Gross Motor Function Measure (GMFM-66), where he is then classified on the Gross Motor Function Classification Scale (GMFCS) as level V (i.e., non-ambulant). Additionally, his ability to use his hands is assessed using the Manual Ability Classification Scale (MACS), and scored as level IV. A prognostic conversation is then planned, and it is compassionately explained at the family meeting that for children who are functioning at GMFCS level V, and are already 5 years of age, walking independently is an extremely unlikely outcome. They explain that children with this level of cerebral palsy typically reach 90% of their motor potential by 3 years of age. The team then explores with the family the hopes they have for Charlie to be able to access his environment in the context of limited independence. The goals are readjusted to include changing position in bed with less assistance and exploring suitable wheeled mobility options to support Charlie's inclusion in all environments.

Scenario 2: Maya is 12 months corrected age with a diagnosis of spastic diplegia. She is able to sit independently for 2 min but is not yet able to transition in and out of sitting. She can take weight through her feet when supported at the trunk. Maya's parents set the goal of independent walking. During her recent therapy appointments, she has been working on automatic balance reactions while supported on an exercise ball. The team use the GMFM-66 to assess Maya and apply the 0–2 criteria of the GMFCS. This indicates that Maya is currently sitting between GMFCS I and II and that while walking independently is a likely medium to long term goal, there are some precursor mobility skills she is likely to attain before independent walking, such as standing independently. The short-term goals are readjusted to include learning to stand independently and cruise at furniture, and transition between sitting and prone, so as scaffold and make progress toward the parent-set longer-term goal of independent walking. With the rehabilitation plan advancing from part-task practice (standing and stepping) to whole task practice (walking).

Goal setting should be an iterative process and adjustments to goals through Layers 1–3 are often required. Layer 1–3 processes can often occur in a single therapeutic conversation or over time dependent on client needs to ensure realistic and motivating goals are set and evaluated. Any adjustments to goals should be reflected in updated and refined wording of goals recorded on the COPM and GAS instruments, so that before and after intervention is measured against the precise goal target.

### READ Model Layer 4: Choose the Evidence-Based Interventions to Meet the Goals

After assessment and realistic goal setting, the next step for healthcare professionals is to sharpen the focus by selecting an intervention/s to meet the goal. To do so, healthcare professionals need to consider: How do I interpret the evidence that is available? Can I apply the published evidence directly to the client in front of me, or are accommodations and adaptations required given the client's personal factors, preferences and comorbidities? Have I considered personal and environmental factors such as family supports and mental health? These are all real and valid questions that affect decision-making.

#### Interpret the Evidence

The Cochrane Central Register of Controlled Trials (CENTRAL) reports more than 100,000 new trials annually and contains almost 1.8 million trials in total (46). The updated systematic review of interventions for children with cerebral palsy identified 182 available published treatment options in 2019 (47), an increase from 64 interventions reported in 2013 (48). There are many additional interventions that parents ask about that have no published evidence, and this is increasingly common with social media and online networks. A challenge exists for healthcare professionals to keep up with the constant and rapid advances in healthcare (49), and to understand which research to trust and which can be applied to their clients.

#### Consider the Intervention's Mechanism of Action and Concordance With the Goal

It is important to consider the purpose of the intervention and the intervention's mechanism of action, and how well the mechanism aligns with the client's goal and context. Often, there is more than one intervention approach that could lead to goal attainment, and sometimes a combination of approaches will augment the treatment effect size. A systematic review of interventions for children with cerebral palsy includes a “A Guide to Interpretation” [(47), p. 12]. The guide encourages healthcare professionals to match the client's goals to the outcome indicator headings in the bubble charts of the systematic review, for example “hand function” or “activity performance” and check the corresponding intervention options with the associated levels of evidence. When choosing interventions for children with cerebral palsy, this guide can help to provide a simplified approach to making sure the intervention is evidence-based with reference to an outcome.

#### Consider the Individual Comorbidities and the Impact on Selection Applicability

Comorbidities are essential to consider as they often moderate the client's success of the chosen intervention. Identifying comorbidities, and a client's strengths, involves obtaining a thorough medical history plus incorporating client preferences with clinical judgement. Previous research on evidence-based decision models focuses on this important relationship between client preferences, clinical judgement and scientific evidence (25). The diagnostic and prognostic observational evidence base will need to be given due consideration in order to personalize the care, since the presence of comorbid is likely to contract the list of appropriate interventions options. For example, comorbidities are common in cerebral palsy, and can be either co-occurring (attributed to the early brain injury) such as epilepsy, intellectual impairment and vision impairment, or secondary complications such as hip dislocation and muscle contracture (50). Vision impairment is a common co-occurring comorbidity for individuals with cerebral palsy, with 1 in 10 experiencing severe vision impairment or blindness (51). For non-verbal clients with cerebral palsy who have a co-existing vision impairment, selection of a communication system with auditory outputs would make use of the client's strengths. Further, for a 10 year old client with dystonic cerebral palsy who wants to work on independence in self-care, Cognitive Orientation to Occupational Performance (CO-OP) might be an appropriate evidence-based intervention (47). However, CO-OP would not be an appropriate selection if the client was non-verbal or was <5 years of age and required a proxy to set their goals. The challenge exists for clinicians and clients to work together to understand the impact of individual comorbidities, in order to ensure goals, activities, and interventions are appropriate, by making use of a client's strengths.

#### Consider the Family's Mental Health and the External Factors

Achievement of goals is influenced by a variety of factors that are individual to the client and their family, including family supports, mental health status and time available to work on goals. The International Classification of Functioning (ICF) system incorporates environmental factors, highlighting the significant role that the physical and social environment can have on function. Physical factors include the individual's physical home/school environment, the local climate, terrain, or building design; social environment factors are attitudes, institutions, and laws; and of particular importance, funding. Decision-making becomes more complex with the addition of these external factors.

### READ Model Layer 5: Choose the Evidence-Based Mode to Match the Intervention Selected

Dependent on the intervention selected, healthcare professionals should consider the various modes of delivery available for this approach, and which might be the most effective. For example, one-to-one vs. group-based intervention, or intervention delivered in the clinic vs. at the client's home, or in their community. Importantly, tele-practice has now been shown to be an effective delivery mode for many interventions (52) and may enable access to services for clients who live in rural and remote areas, as well as promote good infection control in a pandemic.

When weighing-up the most appropriate mode of delivery, healthcare professionals need to consider whether the proposed, or preferred, mode suits the client/their family and the service they work for, and whether supporting evidence of effectiveness exists. If not, the healthcare professional may need to consider whether it is possible to adapt the service delivery model to meet the client's needs or alternatively refer the client to another service that can address their goals.

### READ Model Layer 6: Choose the Evidence-Based Dose to Match the Intervention Selected

To ensure an intervention is effective, an adequate dose (or intensity) must be delivered. Dose or intensity is not a rehabilitation approach in its own right, rather the effective dose is specific to the intervention selected and the mechanisms of action. Given the time and financial costs associated with specific interventions, it is important for the healthcare professional to consider how the recommended dose is going to be achieved both by the family and the healthcare system (34, 43, 53, 54). If funding is not fully available or the client and their family are not in a financial position to contribute to the cost of the intervention, consider selecting the next most effective intervention available to the client. The healthcare professional can also consider a shared care mode of delivery between the healthcare system and the client using a home program. A home program can be used to supplement face-to-face intervention to achieve the recommended dose and reduce the overall financial cost of the intervention, since carers can provide high quality care with training (49). In some circumstances it may not be feasible for the client or family to achieve the recommended dose. When this occurs, it is important for the healthcare professional to acknowledge the disappointment and collectively develop a realistic plan that addresses the current limitations and supports the client and family to access other evidence-based interventions (34, 47).

### READ Model Layer 7: Provide the Intervention and Review the Outcomes and Goals

**Figure 2:**
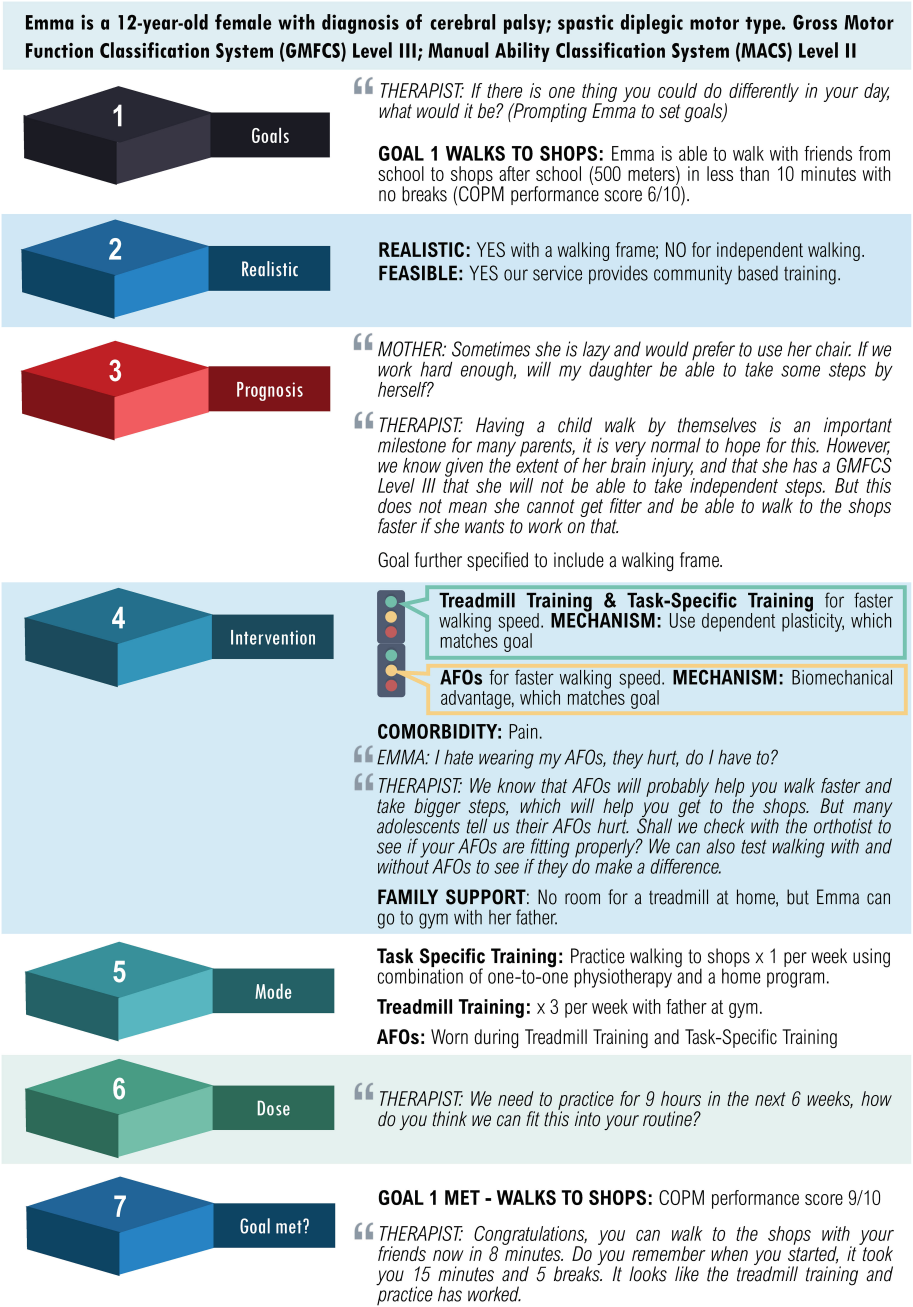
Case study of an adolescent with cerebral palsy using the READ model.

Providing the selected intervention as well as measuring and reviewing intervention outcomes with a client and their family, is a critical step. If the goal has been met, then the client can be discharged, or new goals can be set for another episode of care. If the goal has not been met or goal attainment is unclear, then the selection of the intervention, mode, and dose should be reviewed. In this instance, it is important to consider: if the goal was realistic in the first place; if there are alternate treatments that can be implemented to target the same outcome; and/or if the known effective target dose was achieved. A case study of an adolescent with cerebral palsy is described using the decision-making process of the READ Model (Figure 2).

Further examples of case studies can be found in the Supplementary Material.

## Discussion

The purpose of EBP has always been to improve client care. More recently, the idea has been postulated to integrate health system and service organization as a “fourth factor” into the model to help frame EBP more wholistically (54). Acknowledging the important influence of these often inflexible external factors is hoped to allow clients and families to set more realistic goals, and to understand their outcomes as a result of the multi-layered decision-making process. The READ Model can absorb this fourth factor in Layer 2, “are the goals feasible and realistic”?

A key enabler or barrier to implementation of EBP is the experience and knowledge of the healthcare provider. Staying up to date with evidence is challenging for busy health professionals, however some helpful frameworks exist. Novak et al. (34, 47, 48) use a simple traffic light color coded evidence system to provide useful and comparative information to aid rapid decision-making. The traffic light paper provides a platform for approaching, measuring, and choosing information (rather than a stand-alone advice paper). The color systems are green = “do it” (proven effective), yellow = “probably do it” and red = “don't do it” (proven ineffective or harmful). Yellow coding does not mean “caution,” rather “measure,” because we are uncertain of the estimate of effects, and thus it is important to confirm whether the intervention is helping the person achieve their goal.

In addition to the choice of intervention, it is critical for the healthcare professional to consider the mode of delivery and dose of intervention, and the fit with the service model they work within. There has been an increased focus on intensity or dose of therapy in recent times with an understanding that training-based rehabilitation harness neuroplasticity and improves function. Historically, service delivery models for children with disabilities offer 6-week blocks of weekly therapy. Whilst this is an evidence-based dose for several talk-based psychology interventions, this is not the case for all rehabilitation interventions. For example, Cognitive Behavior Therapy is delivered in 6-week blocks but this is not the recommended evidence-based dose for goal-based training of general functional skills (53). If the service delivery model is under-dosing the known effective intervention, it is the healthcare professional's responsibility to have an open and transparent conversation with the family. This may include exploring shared care models (where part of the dose is provided by the professional and the remainder of the dose is provided by the family via a home program) so that the intervention can be provided at the known effective dose.

In conclusion, evidence-based decision-making is both an art and a science. It involves listening to the client's aspirations and goals in the context of their prognosis, and considering the evidence-based intervention options available as well as the delivery formats and crucial dose needed to achieve them. The READ Model enables wholistic EBP whilst remaining family-centered, goal-based, strengths-based, and solution focused, for the purpose of maximizing improvements that are meaningful to the client.

## Author Contributions

All authors listed have made a substantial, direct and intellectual contribution to the work, and approved it for publication.

## Conflict of Interest

The authors declare that the research was conducted in the absence of any commercial or financial relationships that could be construed as a potential conflict of interest.

## Publisher's Note

All claims expressed in this article are solely those of the authors and do not necessarily represent those of their affiliated organizations, or those of the publisher, the editors and the reviewers. Any product that may be evaluated in this article, or claim that may be made by its manufacturer, is not guaranteed or endorsed by the publisher.

## Supplementary Material

Supplementary Figure 1 Case study of an adolescent with cerebral palsy using the READ model.

Supplementary Figure 2 Case study of an infant with suspected autism spectrum disorder using the READ model.

Supplementary Figure 3 Case study of an infant with suspected autism spectrum disorder using the READ model.
