# Supplementary figures and images for: Rehabilitation Evidence-Based Decision-Making: The READ Model
Source: Front Rehabil Sci. 2021 Oct 5;2:726410. doi: 10.3389/fresc.2021.726410 (PMC9397823; doi:10.3389/fresc.2021.726410)

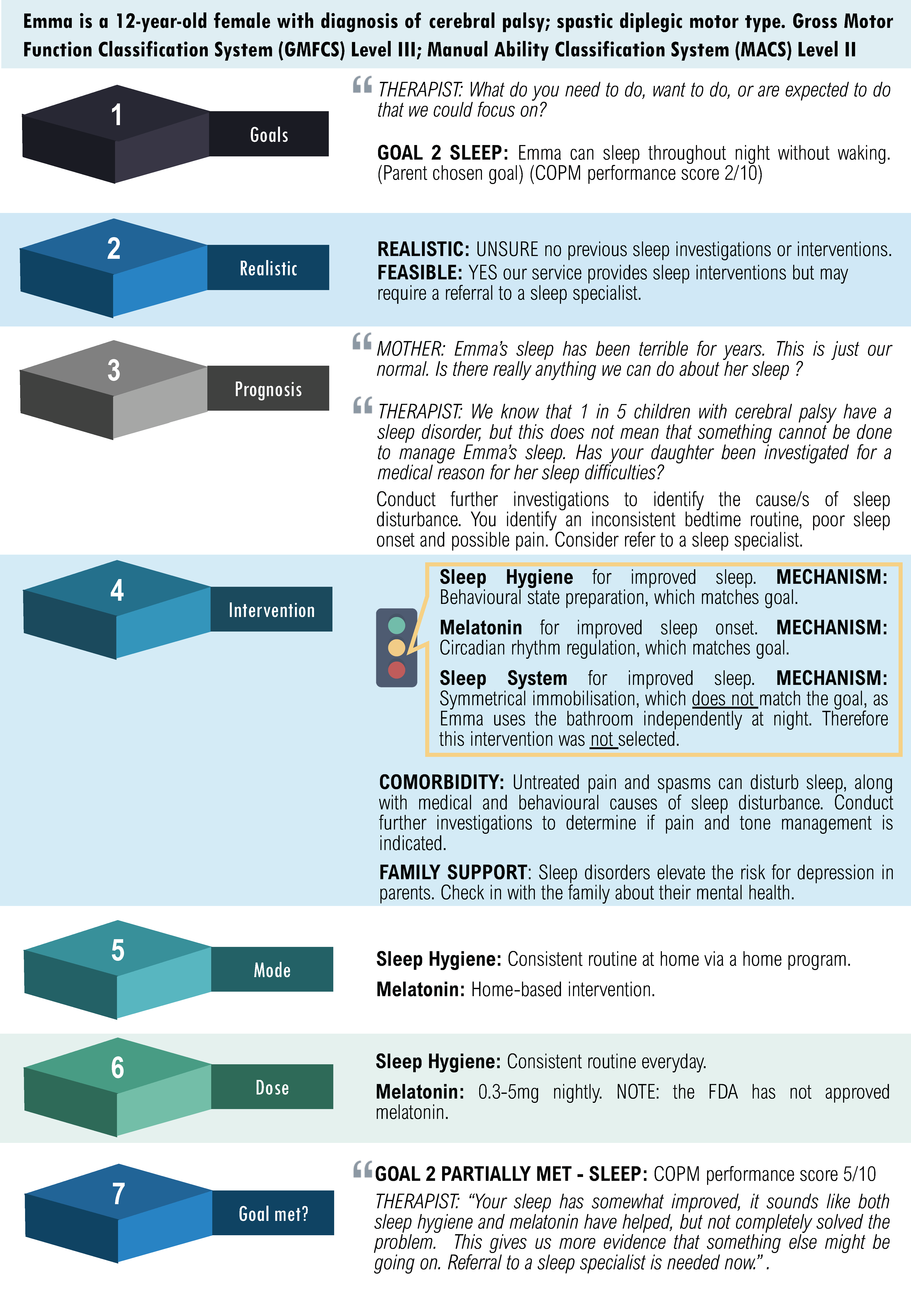

Supplement: Supplementary Figure 1 — Case study of an adolescent with cerebral palsy using the READ model. [file Image_1.TIF]

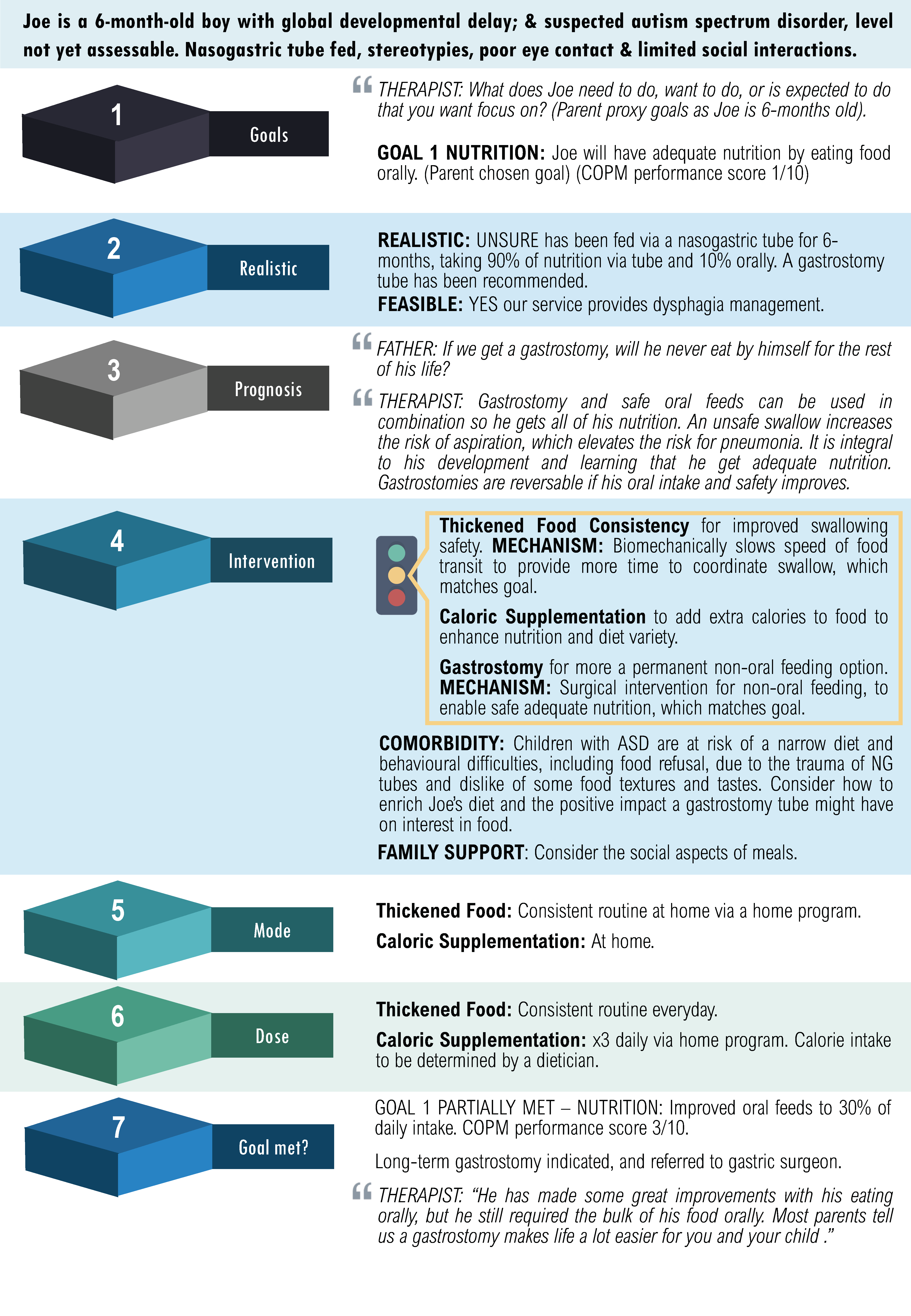

Supplement: Supplementary Figure 2 — Case study of an infant with suspected autism spectrum disorder using the READ model. [file Image_2.TIF]

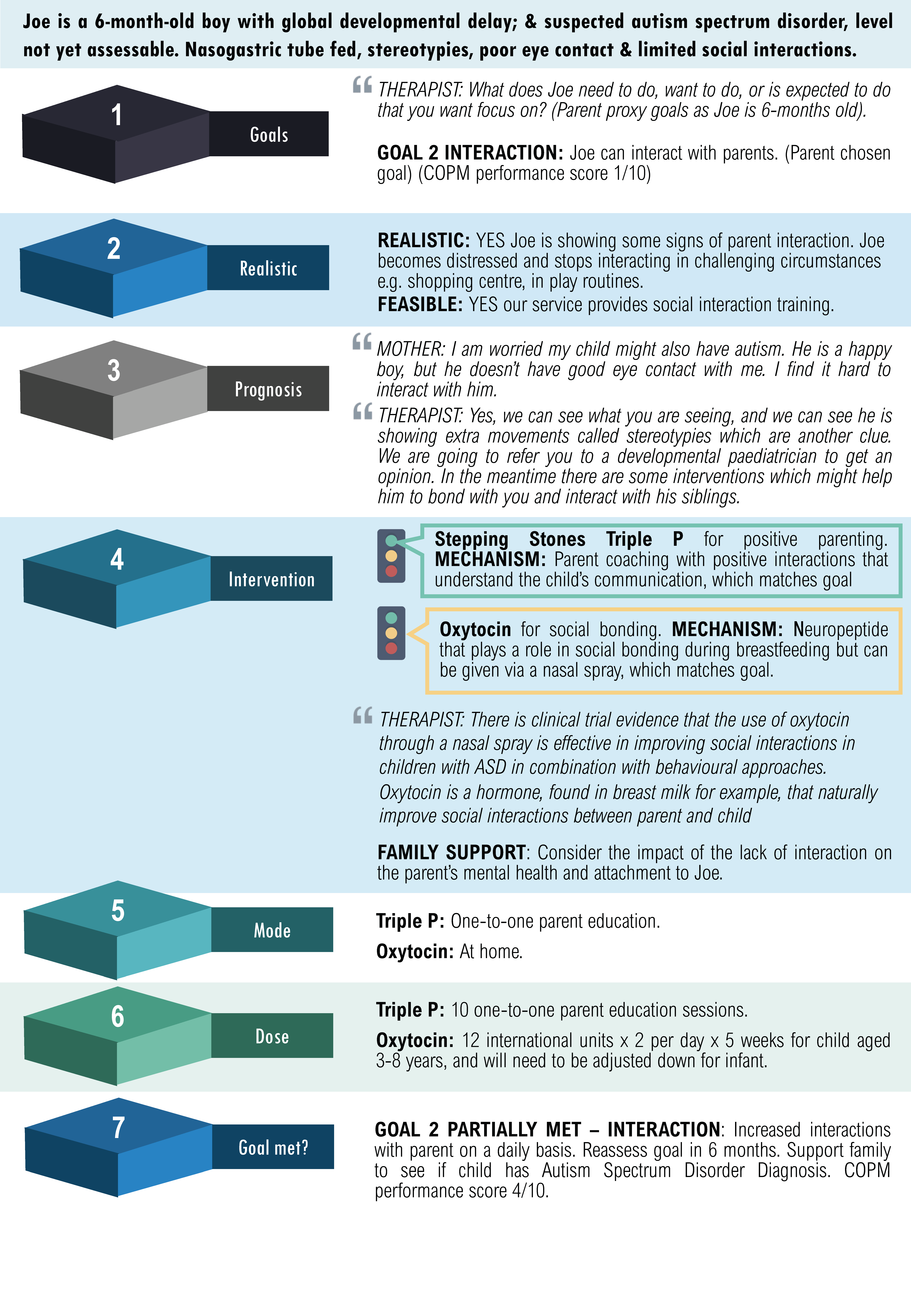

Supplement: Supplementary Figure 3 — Case study of an infant with suspected autism spectrum disorder using the READ model. [file Image_3.TIF]
